# Bis{μ-2-[(*N*,*N*-diisopropyl­carbamothio­yl)sulfan­yl]acetato-κ^2^ *O*:*O*′}bis­(bis­(4-chloro­benz­yl){2-[(*N*,*N*-diisopropyl­carbamothio­yl)sulfan­yl]acetato-κ^2^ *O*,*O*′}tin(IV))

**DOI:** 10.1107/S1600536811015716

**Published:** 2011-05-07

**Authors:** Thy Chun Keng, Kong Mun Lo, Seik Weng Ng

**Affiliations:** a Department of Chemistry, University of Malaya, 50603 Kuala Lumpur, Malaysia

## Abstract

The dinuclear title complex, [Sn_2_(C_7_H_6_Cl)_4_(C_9_H_16_NO_2_S_2_)_4_], lies on a center of inversion. The Sn^IV^ atoms are chelated by one of the two carboxyl­ate ions; the other carboxyl­ate ion bridges two metal atoms. The geometry of the six-coordinate Sn^IV^ atom is a distorted *trans*-C_2_SnO_4_ octa­hedron [C—Sn—C = 155.32 (8)°].

## Related literature

For the direct synthesis of the organotin chloride reactant, see: Sisido *et al.* (1961 ▶). For the synthesis of the carb­oxy­lic acid, see: Nachmias (1952 ▶). For a review of the crystal structures of organotin carboxyl­ates, see: Tiekink (1991 ▶, 1994 ▶).
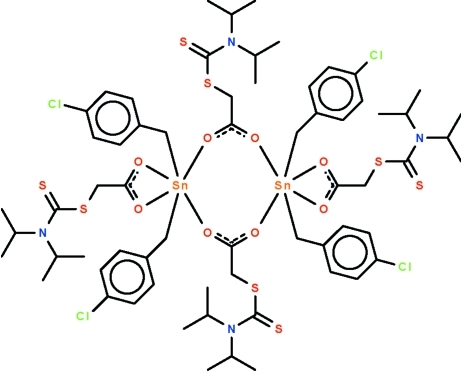


## Experimental

### 

#### Crystal data

[Sn_2_(C_7_H_6_Cl)_4_(C_9_H_16_NO_2_S_2_)_4_]
*M* *_r_* = 1677.04
Triclinic, 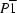

*a* = 11.0257 (1) Å
*b* = 13.1588 (2) Å
*c* = 14.5369 (2) Å
α = 96.4464 (5)°
β = 101.0660 (5)°
γ = 112.8168 (5)°
*V* = 1866.82 (4) Å^3^
*Z* = 1
Mo *K*α radiation
μ = 1.09 mm^−1^
*T* = 100 K
0.25 × 0.20 × 0.15 mm

#### Data collection

Bruker SMART APEX diffractometer
Absorption correction: multi-scan (*SADABS*; Sheldrick, 1996 ▶) *T*_min_ = 0.773, *T*_max_ = 0.854
17479 measured reflections
8479 independent reflections
7912 reflections with *I* > 2σ(*I*)
*R*_int_ = 0.014

#### Refinement

*R*[*F* ^2^ > 2σ(*F* ^2^)] = 0.024
*wR*(*F* ^2^) = 0.083
*S* = 0.93
8479 reflections
406 parameters
H-atom parameters constrained
Δρ_max_ = 1.36 e Å^−3^
Δρ_min_ = −0.40 e Å^−3^

### 

Data collection: *APEX2* (Bruker, 2009 ▶); cell refinement: *SAINT* (Bruker, 2009 ▶); data reduction: *SAINT*; program(s) used to solve structure: *SHELXS97* (Sheldrick, 2008 ▶); program(s) used to refine structure: *SHELXL97* (Sheldrick, 2008 ▶); molecular graphics: *X-SEED* (Barbour, 2001 ▶); software used to prepare material for publication: *publCIF* (Westrip, 2010 ▶).

## Supplementary Material

Crystal structure: contains datablocks global, I. DOI: 10.1107/S1600536811015716/bt5526sup1.cif

Structure factors: contains datablocks I. DOI: 10.1107/S1600536811015716/bt5526Isup2.hkl

Additional supplementary materials:  crystallographic information; 3D view; checkCIF report

## Figures and Tables

**Fig. 1.**
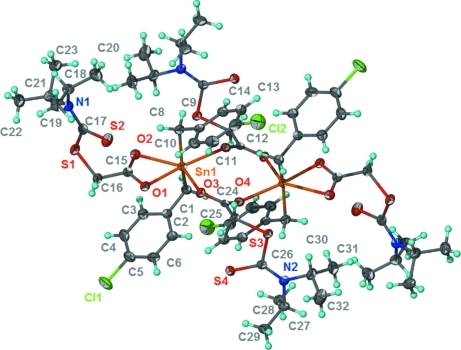
Anisotropic displacement ellipsoid plot (Barbour, 2001) of [Sn(C7H6Cl)2(C9H16NO2S2)2]2 at the 70% probability level; hydrogen atoms are drawn as spheres of arbitrary radius.

## supplementary crystallographic information

### Comment

Diorganotin dicarboxylates are generally six-coordinate compounds as the carboxyl –CO_2_ portion of the anion functions either in a chelating or in a bridging mode (Tiekink, 1991, 1994). The title compound (Scheme I) exists as a centrosymmetric dinuclear molecule in which the anion of one formula unit functions in a chelating mode whereas the other anion functions in a chelating mode (Fig. 1). In dinuclear [Sn(C_7_H_6_Cl)_2_(C_9_H_16_NO_2_S_2_)_2_]_2_, the Sn^IV^ atom is chelated by one of the two carboxylate ions; the other carboxylate ion bridges two metal atoms. The geometry of the six-coordinate Sn^IV^ atom is a *trans*-C_2_SnO_4_ octahedron [C–Sn–C 155.32 (8) °]. The chelation is not isobidentate.

### Experimental

Di(4-chlorobenzyl)tin oxide was prepared by the base hydrolysis of di(4-chlorobenzyl)tin dichloride with 10% sodium hydroxide. The diorganotin dichloride was synthesized by the direct reaction of 4-chlorobenzyl chloride and metallic tin according to a literature procedure (Sisido *et al.*, 1961). The carboxylic acid was synthesized by using literature procedure (Nachmias, 1952). The diorganotin oxide (0.78 g, 2 mmol) and *N,N*-diisopropyldithiocarbamylacetic acid (0.94 g, 2 mmol) were heated in ethanol (100 ml) for an hour until the oxide dissolved. The solution was filtered; slow evaporation of the filtrate gave colorless crystals.

### Refinement

H-atoms were placed in calculated positions (C—H 0.95 to 0.99 Å) and were included in the refinement in the riding model approximation, with *U*(H) set to 1.2 times *U*_eq_(C).

### Crystal data

**Table:**

[Sn_2_(C_7_H_6_Cl)_4_(C_9_H_16_NO_2_S_4_)_2_]	*Z* = 1
*M**_r_* = 1677.04	*F*(000) = 860
Triclinic, *P*1	*D*_x_ = 1.492 Mg m^−^^3^
Hall symbol: -P 1	Mo *K*α radiation, λ = 0.71073 Å
*a* = 11.0257 (1) Å	Cell parameters from 9894 reflections
*b* = 13.1588 (2) Å	θ = 2.5–28.4°
*c* = 14.5369 (2) Å	µ = 1.09 mm^−^^1^
α = 96.4464 (5)°	*T* = 100 K
β = 101.0660 (5)°	Block, colorless
γ = 112.8168 (5)°	0.25 × 0.20 × 0.15 mm
*V* = 1866.82 (4) Å^3^	

### Data collection

**Table:**

Bruker SMART APEX diffractometer	8479 independent reflections
Radiation source: fine-focus sealed tube	7912 reflections with *I* > 2σ(*I*)
graphite	*R*_int_ = 0.014
ω scans	θ_max_ = 27.5°, θ_min_ = 2.2°
Absorption correction: multi-scan (*SADABS*; Sheldrick, 1996)	*h* = −14→14
*T*_min_ = 0.773, *T*_max_ = 0.854	*k* = −17→16
17479 measured reflections	*l* = −18→18

### Refinement

**Table:**

Refinement on *F*^2^	Primary atom site location: structure-invariant direct methods
Least-squares matrix: full	Secondary atom site location: difference Fourier map
*R*[*F*^2^ > 2σ(*F*^2^)] = 0.024	Hydrogen site location: inferred from neighbouring sites
*wR*(*F*^2^) = 0.083	H-atom parameters constrained
*S* = 0.93	*w* = 1/[σ^2^(*F*_o_^2^) + (0.0633*P*)^2^ + 1.4914*P*] where *P* = (*F*_o_^2^ + 2*F*_c_^2^)/3
8479 reflections	(Δ/σ)_max_ = 0.001
406 parameters	Δρ_max_ = 1.36 e Å^−^^3^
0 restraints	Δρ_min_ = −0.40 e Å^−^^3^

### Fractional atomic coordinates and isotropic or equivalent isotropic displacement parameters (Å^2^)

**Table:**

	*x*	*y*	*z*	*U*_iso_*/*U*_eq_	
Sn1	0.406607 (11)	0.336032 (10)	0.363697 (8)	0.01541 (5)	
Cl1	0.79909 (7)	0.41135 (6)	0.03727 (5)	0.04089 (15)	
Cl2	0.49005 (7)	0.19243 (6)	0.83012 (4)	0.04131 (15)	
S1	0.20626 (5)	−0.00123 (4)	0.06705 (3)	0.02075 (10)	
S2	0.28467 (6)	−0.04328 (4)	0.26242 (4)	0.02568 (11)	
S3	0.89452 (5)	0.59366 (4)	0.67643 (3)	0.01785 (10)	
S4	0.96621 (5)	0.63196 (4)	0.49095 (3)	0.01809 (10)	
O1	0.47770 (14)	0.23694 (12)	0.27828 (10)	0.0198 (3)	
O2	0.27222 (14)	0.21691 (12)	0.20565 (10)	0.0199 (3)	
O3	0.59477 (13)	0.37482 (11)	0.46061 (10)	0.0181 (3)	
O4	0.64367 (13)	0.55367 (11)	0.52227 (10)	0.0169 (3)	
N1	0.05428 (17)	−0.17675 (14)	0.12945 (12)	0.0187 (3)	
N2	1.08837 (17)	0.78195 (14)	0.65749 (12)	0.0200 (3)	
C1	0.4573 (2)	0.47745 (16)	0.29490 (14)	0.0191 (4)	
H1A	0.3730	0.4786	0.2574	0.023*	
H1B	0.5066	0.5483	0.3438	0.023*	
C2	0.54474 (19)	0.46880 (16)	0.22990 (14)	0.0184 (4)	
C3	0.4923 (2)	0.43674 (17)	0.13091 (15)	0.0215 (4)	
H3	0.4018	0.4264	0.1037	0.026*	
C4	0.5703 (2)	0.41951 (18)	0.07087 (15)	0.0255 (4)	
H4	0.5337	0.3975	0.0034	0.031*	
C5	0.7014 (2)	0.43497 (18)	0.11127 (17)	0.0264 (4)	
C6	0.7578 (2)	0.46882 (18)	0.20934 (16)	0.0251 (4)	
H6	0.8489	0.4800	0.2360	0.030*	
C7	0.6792 (2)	0.48612 (17)	0.26803 (15)	0.0214 (4)	
H7	0.7175	0.5101	0.3353	0.026*	
C8	0.27651 (19)	0.20341 (16)	0.41890 (14)	0.0192 (4)	
H8A	0.1884	0.2090	0.4126	0.023*	
H8B	0.2584	0.1304	0.3791	0.023*	
C9	0.33225 (19)	0.20376 (15)	0.52141 (14)	0.0177 (4)	
C10	0.4326 (2)	0.16432 (18)	0.54665 (16)	0.0232 (4)	
H10	0.4674	0.1392	0.4982	0.028*	
C11	0.4821 (2)	0.16130 (19)	0.64091 (17)	0.0272 (4)	
H11	0.5506	0.1348	0.6572	0.033*	
C12	0.4302 (2)	0.19759 (18)	0.71133 (16)	0.0258 (4)	
C13	0.3319 (2)	0.23779 (17)	0.68898 (15)	0.0232 (4)	
H13	0.2976	0.2629	0.7377	0.028*	
C14	0.2840 (2)	0.24098 (16)	0.59425 (15)	0.0207 (4)	
H14	0.2169	0.2691	0.5787	0.025*	
C15	0.37035 (19)	0.19069 (16)	0.20849 (14)	0.0185 (4)	
C16	0.3714 (2)	0.10820 (17)	0.12758 (15)	0.0215 (4)	
H16A	0.4317	0.0730	0.1536	0.026*	
H16B	0.4107	0.1503	0.0803	0.026*	
C17	0.1718 (2)	−0.08442 (16)	0.15656 (14)	0.0188 (4)	
C18	0.0199 (2)	−0.26663 (17)	0.18707 (15)	0.0218 (4)	
H18	−0.0696	−0.3276	0.1491	0.026*	
C19	0.1204 (2)	−0.32062 (19)	0.19771 (17)	0.0281 (4)	
H19A	0.1313	−0.3439	0.1345	0.042*	
H19B	0.0858	−0.3866	0.2264	0.042*	
H19C	0.2086	−0.2660	0.2390	0.042*	
C20	−0.0031 (3)	−0.2275 (2)	0.28203 (17)	0.0324 (5)	
H20A	−0.0682	−0.1936	0.2708	0.049*	
H20B	0.0835	−0.1715	0.3244	0.049*	
H20C	−0.0394	−0.2921	0.3121	0.049*	
C21	−0.0494 (2)	−0.19933 (17)	0.03826 (14)	0.0213 (4)	
H21	−0.0217	−0.1286	0.0126	0.026*	
C22	−0.0516 (2)	−0.2910 (2)	−0.03654 (15)	0.0281 (4)	
H22A	0.0401	−0.2707	−0.0456	0.042*	
H22B	−0.1144	−0.2988	−0.0973	0.042*	
H22C	−0.0820	−0.3626	−0.0149	0.042*	
C23	−0.1897 (2)	−0.22522 (19)	0.05571 (16)	0.0263 (4)	
H23A	−0.1834	−0.1640	0.1041	0.039*	
H23B	−0.2219	−0.2960	0.0784	0.039*	
H23C	−0.2537	−0.2323	−0.0042	0.039*	
C24	0.66742 (17)	0.46904 (15)	0.52041 (12)	0.0144 (3)	
C25	0.78688 (18)	0.46587 (15)	0.58999 (14)	0.0176 (3)	
H25A	0.8435	0.4459	0.5526	0.021*	
H25B	0.7510	0.4049	0.6249	0.021*	
C26	0.99540 (18)	0.68054 (16)	0.60723 (14)	0.0167 (3)	
C27	1.1767 (2)	0.87019 (16)	0.61264 (15)	0.0209 (4)	
H27	1.2361	0.9355	0.6667	0.025*	
C28	1.0951 (2)	0.91474 (17)	0.54522 (16)	0.0237 (4)	
H28A	1.0353	0.9356	0.5775	0.036*	
H28B	1.0400	0.8562	0.4876	0.036*	
H28C	1.1575	0.9811	0.5271	0.036*	
C29	1.2744 (2)	0.83578 (19)	0.56923 (18)	0.0282 (5)	
H29A	1.3235	0.8079	0.6162	0.042*	
H29B	1.3397	0.9012	0.5519	0.042*	
H29C	1.2226	0.7762	0.5118	0.042*	
C30	1.1115 (2)	0.81703 (18)	0.76301 (15)	0.0267 (4)	
H30	1.0487	0.7518	0.7848	0.032*	
C31	1.0746 (2)	0.9154 (2)	0.78689 (17)	0.0342 (5)	
H31A	0.9812	0.8960	0.7509	0.051*	
H31B	1.1373	0.9821	0.7694	0.051*	
H31C	1.0818	0.9312	0.8557	0.051*	
C32	1.2568 (3)	0.8413 (2)	0.81602 (17)	0.0349 (5)	
H32A	1.2698	0.8646	0.8849	0.052*	
H32B	1.3215	0.9018	0.7933	0.052*	
H32C	1.2723	0.7732	0.8038	0.052*	

### Atomic displacement parameters (Å^2^)

**Table:**

	*U*^11^	*U*^22^	*U*^33^	*U*^12^	*U*^13^	*U*^23^
Sn1	0.01341 (8)	0.01605 (8)	0.01545 (8)	0.00530 (5)	0.00327 (5)	0.00229 (5)
Cl1	0.0447 (3)	0.0428 (3)	0.0452 (4)	0.0206 (3)	0.0307 (3)	0.0069 (3)
Cl2	0.0425 (3)	0.0562 (4)	0.0249 (3)	0.0218 (3)	0.0028 (2)	0.0132 (3)
S1	0.0221 (2)	0.0181 (2)	0.0158 (2)	0.00386 (18)	0.00156 (18)	0.00244 (17)
S2	0.0279 (3)	0.0224 (2)	0.0224 (2)	0.0108 (2)	−0.0030 (2)	0.00348 (19)
S3	0.0161 (2)	0.0179 (2)	0.0152 (2)	0.00339 (17)	0.00239 (16)	0.00325 (17)
S4	0.0186 (2)	0.0196 (2)	0.0171 (2)	0.00896 (18)	0.00526 (17)	0.00303 (17)
O1	0.0156 (6)	0.0191 (6)	0.0208 (7)	0.0053 (5)	0.0023 (5)	0.0001 (5)
O2	0.0169 (6)	0.0217 (7)	0.0207 (7)	0.0088 (5)	0.0036 (5)	0.0029 (5)
O3	0.0155 (6)	0.0164 (6)	0.0192 (6)	0.0057 (5)	0.0015 (5)	0.0002 (5)
O4	0.0149 (6)	0.0175 (6)	0.0202 (7)	0.0082 (5)	0.0055 (5)	0.0039 (5)
N1	0.0189 (8)	0.0218 (8)	0.0157 (8)	0.0091 (6)	0.0025 (6)	0.0056 (6)
N2	0.0182 (8)	0.0181 (8)	0.0202 (8)	0.0044 (6)	0.0048 (6)	0.0029 (6)
C1	0.0186 (9)	0.0193 (9)	0.0204 (9)	0.0079 (7)	0.0071 (7)	0.0046 (7)
C2	0.0183 (9)	0.0172 (8)	0.0191 (9)	0.0059 (7)	0.0064 (7)	0.0036 (7)
C3	0.0220 (9)	0.0228 (9)	0.0197 (9)	0.0091 (8)	0.0054 (8)	0.0049 (7)
C4	0.0332 (11)	0.0258 (10)	0.0185 (9)	0.0119 (9)	0.0103 (8)	0.0038 (8)
C5	0.0305 (11)	0.0235 (10)	0.0299 (11)	0.0108 (8)	0.0179 (9)	0.0071 (8)
C6	0.0194 (9)	0.0248 (10)	0.0319 (11)	0.0081 (8)	0.0098 (8)	0.0078 (8)
C7	0.0186 (9)	0.0222 (9)	0.0213 (9)	0.0058 (7)	0.0058 (7)	0.0055 (8)
C8	0.0152 (8)	0.0182 (9)	0.0219 (9)	0.0055 (7)	0.0039 (7)	0.0021 (7)
C9	0.0140 (8)	0.0150 (8)	0.0234 (9)	0.0048 (7)	0.0059 (7)	0.0045 (7)
C10	0.0207 (9)	0.0244 (10)	0.0278 (10)	0.0117 (8)	0.0086 (8)	0.0055 (8)
C11	0.0212 (10)	0.0318 (11)	0.0322 (11)	0.0145 (9)	0.0053 (8)	0.0105 (9)
C12	0.0236 (10)	0.0268 (10)	0.0234 (10)	0.0069 (8)	0.0034 (8)	0.0089 (8)
C13	0.0229 (9)	0.0225 (9)	0.0230 (10)	0.0073 (8)	0.0082 (8)	0.0043 (8)
C14	0.0196 (9)	0.0184 (9)	0.0258 (10)	0.0081 (7)	0.0086 (8)	0.0057 (8)
C15	0.0172 (8)	0.0184 (8)	0.0185 (9)	0.0061 (7)	0.0049 (7)	0.0032 (7)
C16	0.0183 (9)	0.0209 (9)	0.0205 (9)	0.0057 (7)	0.0033 (7)	−0.0014 (7)
C17	0.0204 (9)	0.0198 (9)	0.0180 (9)	0.0109 (7)	0.0037 (7)	0.0033 (7)
C18	0.0214 (9)	0.0240 (9)	0.0200 (9)	0.0078 (8)	0.0060 (7)	0.0098 (8)
C19	0.0294 (11)	0.0255 (10)	0.0333 (12)	0.0135 (9)	0.0086 (9)	0.0130 (9)
C20	0.0350 (12)	0.0403 (13)	0.0239 (11)	0.0147 (10)	0.0132 (9)	0.0086 (10)
C21	0.0197 (9)	0.0239 (9)	0.0165 (9)	0.0075 (8)	−0.0003 (7)	0.0049 (7)
C22	0.0313 (11)	0.0325 (11)	0.0186 (10)	0.0131 (9)	0.0043 (8)	0.0018 (8)
C23	0.0192 (9)	0.0289 (10)	0.0283 (11)	0.0096 (8)	0.0022 (8)	0.0048 (9)
C24	0.0112 (7)	0.0167 (8)	0.0145 (8)	0.0039 (6)	0.0052 (6)	0.0032 (7)
C25	0.0144 (8)	0.0145 (8)	0.0198 (9)	0.0037 (7)	0.0010 (7)	0.0021 (7)
C26	0.0141 (8)	0.0184 (8)	0.0194 (9)	0.0084 (7)	0.0044 (7)	0.0051 (7)
C27	0.0182 (9)	0.0179 (9)	0.0263 (10)	0.0056 (7)	0.0083 (8)	0.0059 (8)
C28	0.0235 (10)	0.0179 (9)	0.0310 (11)	0.0076 (8)	0.0101 (8)	0.0086 (8)
C29	0.0215 (10)	0.0270 (10)	0.0415 (13)	0.0123 (8)	0.0145 (9)	0.0093 (9)
C30	0.0259 (10)	0.0228 (10)	0.0185 (10)	−0.0003 (8)	0.0026 (8)	−0.0011 (8)
C31	0.0273 (11)	0.0430 (13)	0.0238 (11)	0.0110 (10)	0.0035 (9)	−0.0072 (10)
C32	0.0375 (13)	0.0333 (12)	0.0269 (11)	0.0144 (10)	−0.0040 (10)	0.0021 (9)

### Geometric parameters (Å, °)

**Table:**

Sn1—O3	2.1083 (13)	C12—C13	1.381 (3)
Sn1—C8	2.136 (2)	C13—C14	1.389 (3)
Sn1—C1	2.1451 (19)	C13—H13	0.9500
Sn1—O1	2.1585 (14)	C14—H14	0.9500
Sn1—O4^i^	2.3661 (13)	C15—C16	1.514 (3)
Sn1—O2	2.4524 (14)	C16—H16A	0.9900
Cl1—C5	1.744 (2)	C16—H16B	0.9900
Cl2—C12	1.745 (2)	C18—C20	1.520 (3)
S1—C17	1.795 (2)	C18—C19	1.525 (3)
S1—C16	1.798 (2)	C18—H18	1.0000
S2—C17	1.663 (2)	C19—H19A	0.9800
S3—C25	1.7945 (19)	C19—H19B	0.9800
S3—C26	1.805 (2)	C19—H19C	0.9800
S4—C26	1.6609 (19)	C20—H20A	0.9800
O1—C15	1.282 (2)	C20—H20B	0.9800
O2—C15	1.252 (2)	C20—H20C	0.9800
O3—C24	1.286 (2)	C21—C22	1.520 (3)
O4—C24	1.238 (2)	C21—C23	1.528 (3)
O4—Sn1^i^	2.3661 (13)	C21—H21	1.0000
N1—C17	1.336 (3)	C22—H22A	0.9800
N1—C21	1.489 (2)	C22—H22B	0.9800
N1—C18	1.497 (2)	C22—H22C	0.9800
N2—C26	1.341 (2)	C23—H23A	0.9800
N2—C30	1.494 (3)	C23—H23B	0.9800
N2—C27	1.503 (2)	C23—H23C	0.9800
C1—C2	1.499 (3)	C24—C25	1.517 (2)
C1—H1A	0.9900	C25—H25A	0.9900
C1—H1B	0.9900	C25—H25B	0.9900
C2—C3	1.392 (3)	C27—C28	1.521 (3)
C2—C7	1.397 (3)	C27—C29	1.529 (3)
C3—C4	1.396 (3)	C27—H27	1.0000
C3—H3	0.9500	C28—H28A	0.9800
C4—C5	1.377 (3)	C28—H28B	0.9800
C4—H4	0.9500	C28—H28C	0.9800
C5—C6	1.386 (3)	C29—H29A	0.9800
C6—C7	1.387 (3)	C29—H29B	0.9800
C6—H6	0.9500	C29—H29C	0.9800
C7—H7	0.9500	C30—C31	1.523 (3)
C8—C9	1.498 (3)	C30—C32	1.529 (3)
C8—H8A	0.9900	C30—H30	1.0000
C8—H8B	0.9900	C31—H31A	0.9800
C9—C14	1.395 (3)	C31—H31B	0.9800
C9—C10	1.400 (3)	C31—H31C	0.9800
C10—C11	1.385 (3)	C32—H32A	0.9800
C10—H10	0.9500	C32—H32B	0.9800
C11—C12	1.389 (3)	C32—H32C	0.9800
C11—H11	0.9500		
			
O3—Sn1—C8	99.59 (6)	S2—C17—S1	119.19 (12)
O3—Sn1—C1	101.45 (6)	N1—C18—C20	112.71 (18)
C8—Sn1—C1	155.32 (8)	N1—C18—C19	112.50 (16)
O3—Sn1—O1	81.59 (5)	C20—C18—C19	113.09 (18)
C8—Sn1—O1	97.84 (7)	N1—C18—H18	105.9
C1—Sn1—O1	97.86 (6)	C20—C18—H18	105.9
O3—Sn1—O4^i^	89.62 (5)	C19—C18—H18	105.9
C8—Sn1—O4^i^	81.34 (6)	C18—C19—H19A	109.5
C1—Sn1—O4^i^	86.03 (6)	C18—C19—H19B	109.5
O1—Sn1—O4^i^	170.93 (5)	H19A—C19—H19B	109.5
O3—Sn1—O2	138.32 (5)	C18—C19—H19C	109.5
C8—Sn1—O2	85.30 (6)	H19A—C19—H19C	109.5
C1—Sn1—O2	87.52 (6)	H19B—C19—H19C	109.5
O1—Sn1—O2	56.78 (5)	C18—C20—H20A	109.5
O4^i^—Sn1—O2	131.86 (5)	C18—C20—H20B	109.5
C17—S1—C16	101.28 (10)	H20A—C20—H20B	109.5
C25—S3—C26	102.78 (9)	C18—C20—H20C	109.5
C15—O1—Sn1	97.25 (12)	H20A—C20—H20C	109.5
C15—O2—Sn1	84.57 (11)	H20B—C20—H20C	109.5
C24—O3—Sn1	124.97 (12)	N1—C21—C22	111.51 (17)
C24—O4—Sn1^i^	134.92 (12)	N1—C21—C23	111.22 (17)
C17—N1—C21	122.40 (16)	C22—C21—C23	112.33 (18)
C17—N1—C18	122.23 (16)	N1—C21—H21	107.2
C21—N1—C18	115.34 (16)	C22—C21—H21	107.2
C26—N2—C30	122.54 (17)	C23—C21—H21	107.2
C26—N2—C27	123.08 (17)	C21—C22—H22A	109.5
C30—N2—C27	114.38 (16)	C21—C22—H22B	109.5
C2—C1—Sn1	109.47 (13)	H22A—C22—H22B	109.5
C2—C1—H1A	109.8	C21—C22—H22C	109.5
Sn1—C1—H1A	109.8	H22A—C22—H22C	109.5
C2—C1—H1B	109.8	H22B—C22—H22C	109.5
Sn1—C1—H1B	109.8	C21—C23—H23A	109.5
H1A—C1—H1B	108.2	C21—C23—H23B	109.5
C3—C2—C7	118.34 (18)	H23A—C23—H23B	109.5
C3—C2—C1	121.19 (18)	C21—C23—H23C	109.5
C7—C2—C1	120.35 (18)	H23A—C23—H23C	109.5
C2—C3—C4	121.21 (19)	H23B—C23—H23C	109.5
C2—C3—H3	119.4	O4—C24—O3	124.49 (17)
C4—C3—H3	119.4	O4—C24—C25	122.89 (16)
C5—C4—C3	118.7 (2)	O3—C24—C25	112.62 (15)
C5—C4—H4	120.6	C24—C25—S3	115.96 (13)
C3—C4—H4	120.6	C24—C25—H25A	108.3
C4—C5—C6	121.64 (19)	S3—C25—H25A	108.3
C4—C5—Cl1	119.24 (18)	C24—C25—H25B	108.3
C6—C5—Cl1	119.12 (17)	S3—C25—H25B	108.3
C5—C6—C7	118.9 (2)	H25A—C25—H25B	107.4
C5—C6—H6	120.5	N2—C26—S4	126.15 (15)
C7—C6—H6	120.5	N2—C26—S3	114.39 (14)
C6—C7—C2	121.11 (19)	S4—C26—S3	119.44 (11)
C6—C7—H7	119.4	N2—C27—C28	112.35 (16)
C2—C7—H7	119.4	N2—C27—C29	113.67 (16)
C9—C8—Sn1	114.60 (13)	C28—C27—C29	113.65 (18)
C9—C8—H8A	108.6	N2—C27—H27	105.4
Sn1—C8—H8A	108.6	C28—C27—H27	105.4
C9—C8—H8B	108.6	C29—C27—H27	105.4
Sn1—C8—H8B	108.6	C27—C28—H28A	109.5
H8A—C8—H8B	107.6	C27—C28—H28B	109.5
C14—C9—C10	118.02 (19)	H28A—C28—H28B	109.5
C14—C9—C8	121.19 (17)	C27—C28—H28C	109.5
C10—C9—C8	120.77 (18)	H28A—C28—H28C	109.5
C11—C10—C9	121.24 (19)	H28B—C28—H28C	109.5
C11—C10—H10	119.4	C27—C29—H29A	109.5
C9—C10—H10	119.4	C27—C29—H29B	109.5
C10—C11—C12	119.10 (19)	H29A—C29—H29B	109.5
C10—C11—H11	120.5	C27—C29—H29C	109.5
C12—C11—H11	120.5	H29A—C29—H29C	109.5
C13—C12—C11	121.1 (2)	H29B—C29—H29C	109.5
C13—C12—Cl2	119.33 (17)	N2—C30—C31	111.48 (18)
C11—C12—Cl2	119.54 (17)	N2—C30—C32	110.90 (19)
C12—C13—C14	119.1 (2)	C31—C30—C32	112.58 (19)
C12—C13—H13	120.5	N2—C30—H30	107.2
C14—C13—H13	120.5	C31—C30—H30	107.2
C13—C14—C9	121.42 (19)	C32—C30—H30	107.2
C13—C14—H14	119.3	C30—C31—H31A	109.5
C9—C14—H14	119.3	C30—C31—H31B	109.5
O2—C15—O1	121.24 (18)	H31A—C31—H31B	109.5
O2—C15—C16	121.85 (17)	C30—C31—H31C	109.5
O1—C15—C16	116.83 (17)	H31A—C31—H31C	109.5
C15—C16—S1	114.07 (14)	H31B—C31—H31C	109.5
C15—C16—H16A	108.7	C30—C32—H32A	109.5
S1—C16—H16A	108.7	C30—C32—H32B	109.5
C15—C16—H16B	108.7	H32A—C32—H32B	109.5
S1—C16—H16B	108.7	C30—C32—H32C	109.5
H16A—C16—H16B	107.6	H32A—C32—H32C	109.5
N1—C17—S2	126.05 (15)	H32B—C32—H32C	109.5
N1—C17—S1	114.75 (14)		
			
O3—Sn1—O1—C15	175.44 (12)	C12—C13—C14—C9	0.5 (3)
C8—Sn1—O1—C15	76.84 (12)	C10—C9—C14—C13	−0.9 (3)
C1—Sn1—O1—C15	−84.06 (12)	C8—C9—C14—C13	177.72 (18)
O2—Sn1—O1—C15	−2.27 (11)	Sn1—O2—C15—O1	−3.81 (18)
O3—Sn1—O2—C15	−1.10 (14)	Sn1—O2—C15—C16	179.68 (18)
C8—Sn1—O2—C15	−100.24 (12)	Sn1—O1—C15—O2	4.3 (2)
C1—Sn1—O2—C15	103.39 (12)	Sn1—O1—C15—C16	−178.97 (14)
O1—Sn1—O2—C15	2.31 (11)	O2—C15—C16—S1	−33.0 (2)
O4^i^—Sn1—O2—C15	−174.20 (10)	O1—C15—C16—S1	150.36 (15)
C8—Sn1—O3—C24	−115.23 (15)	C17—S1—C16—C15	−70.97 (16)
C1—Sn1—O3—C24	51.79 (15)	C21—N1—C17—S2	171.63 (15)
O1—Sn1—O3—C24	148.18 (15)	C18—N1—C17—S2	−10.5 (3)
O4^i^—Sn1—O3—C24	−34.08 (14)	C21—N1—C17—S1	−9.1 (2)
O2—Sn1—O3—C24	151.06 (13)	C18—N1—C17—S1	168.77 (14)
O3—Sn1—C1—C2	71.86 (14)	C16—S1—C17—N1	−177.62 (15)
C8—Sn1—C1—C2	−140.16 (17)	C16—S1—C17—S2	1.75 (14)
O1—Sn1—C1—C2	−11.10 (14)	C17—N1—C18—C20	68.3 (2)
O4^i^—Sn1—C1—C2	160.66 (13)	C21—N1—C18—C20	−113.7 (2)
O2—Sn1—C1—C2	−67.08 (13)	C17—N1—C18—C19	−61.0 (2)
Sn1—C1—C2—C3	104.94 (18)	C21—N1—C18—C19	116.98 (19)
Sn1—C1—C2—C7	−71.1 (2)	C17—N1—C21—C22	105.8 (2)
C7—C2—C3—C4	1.5 (3)	C18—N1—C21—C22	−72.1 (2)
C1—C2—C3—C4	−174.57 (19)	C17—N1—C21—C23	−127.9 (2)
C2—C3—C4—C5	−0.1 (3)	C18—N1—C21—C23	54.1 (2)
C3—C4—C5—C6	−1.1 (3)	Sn1^i^—O4—C24—O3	118.97 (18)
C3—C4—C5—Cl1	178.89 (16)	Sn1^i^—O4—C24—C25	−60.7 (2)
C4—C5—C6—C7	0.8 (3)	Sn1—O3—C24—O4	−7.9 (3)
Cl1—C5—C6—C7	−179.18 (16)	Sn1—O3—C24—C25	171.81 (11)
C5—C6—C7—C2	0.7 (3)	O4—C24—C25—S3	−0.3 (2)
C3—C2—C7—C6	−1.8 (3)	O3—C24—C25—S3	179.99 (13)
C1—C2—C7—C6	174.30 (18)	C26—S3—C25—C24	−77.30 (15)
O3—Sn1—C8—C9	22.00 (14)	C30—N2—C26—S4	−177.53 (15)
C1—Sn1—C8—C9	−126.20 (18)	C27—N2—C26—S4	3.2 (3)
O1—Sn1—C8—C9	104.73 (14)	C30—N2—C26—S3	3.3 (2)
O4^i^—Sn1—C8—C9	−66.14 (13)	C27—N2—C26—S3	−175.90 (14)
O2—Sn1—C8—C9	160.25 (14)	C25—S3—C26—N2	−178.38 (14)
Sn1—C8—C9—C14	104.11 (18)	C25—S3—C26—S4	2.42 (13)
Sn1—C8—C9—C10	−77.3 (2)	C26—N2—C27—C28	66.6 (2)
C14—C9—C10—C11	0.5 (3)	C30—N2—C27—C28	−112.7 (2)
C8—C9—C10—C11	−178.15 (19)	C26—N2—C27—C29	−64.3 (2)
C9—C10—C11—C12	0.4 (3)	C30—N2—C27—C29	116.4 (2)
C10—C11—C12—C13	−0.9 (3)	C26—N2—C30—C31	−115.4 (2)
C10—C11—C12—Cl2	179.14 (17)	C27—N2—C30—C31	63.9 (2)
C11—C12—C13—C14	0.5 (3)	C26—N2—C30—C32	118.3 (2)
Cl2—C12—C13—C14	−179.55 (16)	C27—N2—C30—C32	−62.4 (2)

Symmetry codes: (i) −*x*+1, −*y*+1, −*z*+1.
